# Exploring the underlying mechanisms of customers’ intention to adopt product recommendations from live streamers: A moderated mediation approach

**DOI:** 10.1371/journal.pone.0314682

**Published:** 2025-02-13

**Authors:** Liping Zhang, Xueping Wu

**Affiliations:** School of Economics and Trade, Fujian Jiangxia University, Fuzhou, China; SGH Warsaw School of Economics: Szkola Glowna Handlowa w Warszawie, POLAND

## Abstract

Live streaming has emerged as one of the indispensable channels for product information dissemination. Product recommendations from live streamers play an increasingly important role in customers’ purchasing decisions. Our study aims to understand the mechanism behind individuals’ intention to adopt product recommendations in live streaming. To that end, we implemented a moderated mediation model to test the direct and indirect effects of perceived value on customers’ intention to adopt product recommendations from live streamers, the mediating role of perceived credibility in the relationship between perceived value and adoption intention, the moderating role of sense of telepresence in the link between perceived value and perceived credibility, and the moderating role of self-identification in the association between perceived credibility and adoption intention. Results show that perceived credibility plays a partial intermediary role in the link between perceived value and adoption intention, sense of telepresence can positively predict perceived credibility but cannot significantly moderate the relationship between perceived value and adoption intention, and self-identification can positively buffer the association between perceived credibility and adoption intention, but negatively moderate the relationship between perceived value and adoption intention. This study advances theoretical research on product recommendations in the live-streaming context and provides practical inspiration for live streamers and managers of social commerce companies.

## Introduction

With the advancement of information technology and the ubiquitous usage of mobile devices, live streaming has been developing rapidly in recent years and has become a new trend worldwide [1, 2]. According to a report published by China Internet Network Information Center, live-streaming users in China has reached 716 million by 2022, accounting for 68.1% of China’s whole internet population [3]. The number of people watching live streaming is growing every year, and the average length of time users spend watching live streaming is also rising steadily [3]. Some accounts of Douyin (the Chinese version of TikTok) attract more than 10 million followers. Live streaming has become one of the main channels for users to obtain information. Live streaming can create virtual situations similar to "face-to-face" scenes for viewers to communicate with streamers in real-time, and enables two-way communication, which can strengthen the sense of presence and enhance immediacy, interactivity, and user involvement [4]. Another salient feature of live streaming is that it can help users collect and disseminate information conveniently. The amount of information is growing explosively on the internet every day, and consumers often become confused when faced with the information [5]. In live streaming, users can obtain selected and targeted information easily. Consequently, the traditional way of product information dissemination and the way customers receive product information are inevitably affected by live streaming.

In traditional e-commerce shopping, consumers obtain product information through advertisements or shopping search engines. These ways of receiving product information have some limitations. First of all, consumers cannot participate in the process of product information dissemination. Second, consumers cannot find a fast and effective method to evaluate the quality of products. Third, when faced with a great quantity of product choices, consumers are often unable to decide which product to choose, and they are also concerned that the product information publicized is exaggerated or untrue. EWOM (electronic word-of-mouth) can provide consumers with certain reference information for online shopping [6]. However, consumers are aware that eWOM also has problems, such as false eWOM manipulated by merchants. About 15% of eWOM are directly or indirectly manipulated by manufacturers [7]. As consumers become increasingly aware of the existence of fake eWOM, product recommendations from other consumers play a growingly important role. Product recommendations are often from people who personally purchase or use the products. When consumers lack first-hand, reliable product information or are confused about product selection, it is reasonable to choose products recommended by fellow customers. Individuals who use others’ shopping experiences as guidance can eliminate wrong choices. Live streaming, through all-round, three-dimensional display and explanation of the products, and interaction with viewers, can allow viewers to have an intuitive understanding of the recommended products [8], make them immersed in the product introduction, and learn more about the details of the products. Live streaming can also enable streamers to illustrate how products are produced and used [9], thus enabling viewers to evaluate the recommended products more comprehensively. What is more, live streaming can allow streamers to reveal their faces and personalities, and communicate with viewers in real-time, which can affect viewers’ adoption psychology towards product recommendations.

As live streaming continues to gain popularity, consumer purchase behavior in the live-streaming environment has garnered increasing attention [10]. A key area of research has been the factors affecting viewers’ purchasing intentions in live streaming. Some scholars believe that the match-up plays a significant role in consumers’ willingness to purchase in live streaming. For instance, Zhang et al. found that streamer-product fit and self-product fit can increase affective intensity and reduce perceived product risk for viewers, thereby enhancing their purchasing intentions during live streaming [11]. Shang et al. believed that background fitting in live streaming could significantly affect customers’ purchase intention. While anchor-background fit could positively impact consumers’ perceived trust, product-background fit could enhance their perceived value [12]. Park et al. discovered that product-source fit affects perceived source attractiveness and trustworthiness, product-content fit influences utilitarian and hedonic attitudes toward the content, and self-product fit leads to higher purchase intentions [13]. Some other researchers believe that the characteristic of anchors plays a vital role in viewers’ intentions to buy products presented during live streams. Central to this inquiry, scholars have identified that factors like charm, expertise, humor, and passion of live streamers are crucial drivers influencing viewers’ intentions to purchase in live streaming [14, 15]. There are also scholars who have examined the impact of the view-streamer relationship on consumer purchasing behavior during live streams. As an example, Ko found that parasocial relationships with streamers play a crucial role in increasing consumers’ willingness to buy during live streams [16]. The emotional attachment of live stream viewers to streamers plays a crucial role in their purchasing decisions during live streams [17, 18]. The above literature review reveals that existing research has sufficiently explored the psychological mechanisms behind customers’ purchase intention in the live-streaming context. However, there is still a lack of studies focusing on the topic of customers’ intention to adopt product recommendations from live streamers. The underlying mechanisms of customers’ intention to adopt product recommendations from live streamers are different from those of customers’ intention to purchase during live streams. On the one hand, consumers’ adoption of product recommendations is a precursor to their purchasing of the recommended products. When viewers adopt product recommendations from a streamer, they might purchase the recommended products immediately, consider buying them in the future, or just recommend the products to their peers. On the other hand, the factors influencing the willingness to adopt product recommendations from streamers may differ from those affecting viewers’ purchase intentions in live streams. The adoption of product recommendations from streamers is mainly influenced by factors like the value of the recommendation, its credibility, and so on. However, viewers’ purchase intentions during live streams can be influenced by a variety of other factors, such as their tendencies toward impulse buying, the time pressure of promotions, etc.

Previous research on product recommendations primarily focused on product recommendations in traditional non-live environments. For instance, by conducting a behavioral experiment, Eisend and Langner found that source attributes, such as source attractiveness, have an impact on the persuasive effectiveness of product recommendation information in traditional non-live environments [19]. Gong and Li discovered that the match between product recommender and product could have a positive influence on consumers’ attitudes toward the product information [20]. There are also scholars who found that the size of the followers could affect the persuasive effectiveness of product information [21]. Given the differences between the live-streaming setting and the traditional product information dissemination environment, investigating how product information is adopted by customers in the live-streaming environment bears certain theoretical and practical significance.

The contributions of this study are three-fold: First, while there are plenty of studies on consumers’ adoption mechanisms of eWOM, customers’ adoption psychology of product recommendations from live streamers has received little scholarly attention. Product recommendations from live streamers have changed the way consumers access product information. Consequently, to explore the mechanisms underlying consumers’ adoption of product recommendations from streamers is of necessity. This study examines the relationships among perceived value, perceived credibility, sense of telepresence, self-identification, and adoption intention, aiming to present a comprehensive model for analyzing customers’ intention to adopt product recommendations from live streamers. Second, this study examines how self-identification with streamers moderates the relationships between perceived credibility and adoption intention, as well as between perceived value and adoption intention. Previously, studies in customer behavior research addressing self-identification focused on self-identification with brands or with tourist sites [22, 23]. This study provides evidence of the importance of self-identification to information adoption intention in the online environment, thereby extending the scarce research that exists in this context. Third, this study explores how perceived credibility mediates the relationship between perceived value and adoption intention, and investigates the moderating role of sense of telepresence in the connection between perceived value and perceived credibility. By drawing on data collected from viewers of live-streaming platforms in China, the current investigation enhances existing understanding of the effects of sense of telepresence and perceived credibility on the decision-making process of online customers.

## Theoretical foundation and hypotheses development

### Value-intention framework

Dodds and Monroe introduced the value-intention framework in 1985, positing that an individual’s inclination to engage in a specific behavior is directly shaped by perceived value of behavior consequences [24]. Perceived value is defined as consumers’ overall assessment of the utility of an object, grounded in perceptions of what is received and what is given [25]. Perceived value stems from equity theory, which represents the trade-off between the benefits or quality customers gain, and the costs they bear, including financial, energy, time, and cognitive costs during the evaluation, acquisition, and utilization of a product or service [26]. Enhancing the perceived value of an object can be achieved by either augmenting the benefits it offers or diminishing the sacrifices linked to its acquisition and utilization [27].

The relationship between perceived value and customers’ behavioral intention has been discussed in marketing research for a long time. Williams et al. examined the association between perceived value and individuals’ behavioral intentions in the tourist industry and discovered that the way perceived value affects the behavioral intentions of tourists from different cultural backgrounds varies [28]. Yang and Mattila also found that in the case of luxury restaurants, perceived value has a positive impact on customers’ intentions to visit [29]. The value-intention framework was also frequently used to investigate customers’ online behavioral intentions, such as intention to participate in online group buying [30] and continuous-use intention [31].

Some researchers posited that perceived credibility could jointly influence customers’ behavioral intentions with perceived value. For instance, by investigating the underlying mechanism of how influencer marketing communication affects consumers via social media, Lou and Yuan discovered that the perceived value of product information, together with the perceived credibility, can jointly affect the behavioral intentions of social media users [32]. After exploring the role of influencers in adolescents’ consumption behaviors, Lou and Kim also found that the perceived value of information, together with perceived credibility could exert influences on the online purchasing intention of teenagers [33]. Chen and Ling discovered that the perceived credibility of advertising information, along with overall perceived credibility, impacts consumers’ product evaluation and purchase intentions [34]. Alrwashdeh’s research further confirms the mediating role of perceived credibility in the value-intention relationship by examining how social media influencers influence followers’ patronage intentions [35].

The above literature confirms the applicability of the value-intention framework in the research on customer online behaviors and verifies the mediating role of perceived credibility in the value-intention relationship. Based on previous studies, we developed a moderated mediation model to investigate the underlying mechanisms of customers’ intention to adopt product recommendations in the live-streaming context.

### Perceived value of product recommendations

Perceived value is defined as individuals’ assessment of the value that has been created for them by a supplier given the trade-off between all relevant benefits and sacrifices in a specific use situation [36]. Perceived value has been acknowledged as one of the most important concepts for understanding the behaviors of customers [37, 38]. Perceived value has proven to be a stable predictor of customer behavior [39, 40]. As the theory of consumption values suggests, the motivation of consumers to participate in a specific activity depends on the value they expect to receive [41]. When customers believe that they can receive value from an activity, they will be likely to participate in that activity.

Some researchers pointed out that perceived value should be viewed as a multi-faceted notion, mainly consisting of utilitarian and hedonic domains [42]. "Task-related" and "rational" are two terms often used to describe utilitarian value [43]. Utilitarian value refers to a comprehensive appraisal of functional and instrumental worth based on benefits and sacrifices [44]. Utilitarian value incorporates more cognitive aspects of perception, such as efficiency, convenience, and value-for-money features [22]. Consumers make behavioral decisions based on their rational judgment about the items or services. Compared with offline shopping, traditional e-commerce can save time on information searches, but the volume of information that can be searched is massive, and consumers will be confused about which one to choose. Product recommendations can save the cost of information search and product comparison. In many cases, the products recommended by streamers are ones with which the streamers have had positive experiences. The recommended products may be highly cost-effective or of good quality. Choosing the products recommended by live streamers can often save time, energy, and money. Hedonic value, in contrast to utilitarian value, is more subjective and personal. This sort of value is emotional and experiential, originating more from pleasure and well-being than from mission accomplishment [45]. By watching streamers’ product information sharing and adopting product recommendations of streamers, viewers may meet their emotional needs and alleviate anxiety.

Before viewers adopt product recommendations from live streamers, they will evaluate the perceived value of product recommendations. If they determine that the benefits of adopting the recommendations outweigh the sacrifices, they are likely to adopt them. Perceived economic value, perceived improvement in shopping efficiency, perceived entertainment, among others, are important dimensions for consumers to evaluate the values of product recommendations. Many studies have shown that individuals often have positive attitudes towards information or goods with high perceived value [46]. Bamberg believed that users’ subjective value of the expected outcome determines their attitudes toward the behavior [47]. Liu and Zhang also found that individuals’ intention to adopt product information is positively affected by their perceived value [48]. By studying the role of perceived value on consumers’ attitudinal and behavioral outcomes, Pura proved the positive effects of perceived value on perceived credibility [39]. Lai also discovered that perceived value can positively affect perceived credibility and commitment by studying the behavior patterns of consumers in choosing travel agencies [49]. Thus, we believe that a high level of perceived value of product recommendations will lead to higher perceived credibility and stronger adoption intention. The following hypotheses are then suggested:

Hypothesis 1 (H1). The relationship between perceived value and perceived credibility is positive.
Hypothesis 2 (H2). The relationship between perceived value and intention to adopt product recommendations from live streamers is positive.

### Moderating role of sense of telepresence

Telepresence is defined as a binary experience where one’s sense of self-location and action capabilities are tied to a mediated spatial environment, with mental faculties limited by this mediated environment instead of the actual reality [50]. The term sense of telepresence is used to characterize the experience of a consumer being physically present in a virtual shopping environment [51]. Online video, music, and animation can be utilized to create a high level of sense of telepresence by increasing the level of vividness and user immersion [52]. Individuals typically rely on physical clues to form inferences before really seeing an item. Hence, sense of telepresence is vital in helping customers make choices in the online world [53]. The sensory sense of "being there" may be created using signals from a media interface [54]. Individuals interact with the online environment using their senses, and the degree to which a specific medium can duplicate this sensory information determines the level of telepresence [55].

It is easy for viewers to obtain a large volume of sound, visual, and other kinds of information in real-time in the live-streaming environment. Sense of telepresence, which allows people to completely immerse themselves in a virtual environment despite distance and geographical constraints, may provoke a strong emotional response [56]. A person who is immersed in the environment can show better cognitive absorption and concentrated attention [57]. Sense of telepresence can enhance customer evaluation [58]. When an individual is immersed in the live-streaming environment, he or she may concentrate on the details of the product and product recommendations. When an individual has a better understanding of the product and the product recommendation, he or she can perceive the value of the product and the product recommendation better [59]. Hence, viewers’ interest in the product and product recommendations will increase accordingly.

In a typical business transaction involving a product, consumers often feel compelled to see and evaluate it in person before making buying decisions [60]. While traditional e-commerce environments do not allow for in-person product evaluation, telepresence has proven to be an efficacious way to give people the opportunity to direct experience [61]. A strong sense of telepresence may be created when a medium draws objects near to viewers, allowing them to indirectly interact with the objects [62]. The immediate perceptual feeling of "being in the mediated environment" and the telepresence experience may strengthen the effectiveness of persuasion [63].

As we discussed before, perceived value is a main predictor of perceived credibility [46, 49]. Cheon believed that sense of telepresence can work with perceived value to influence viewers’ perceived credibility of online information [64]. Viewers with a stronger feeling of telepresence may be able to have a better understanding of the product and the product recommendation. As a consequence, the perceived uncertainty may be reduced, and consumers can be more confident in making adoption decisions [65]. A more open and transparent purchasing environment gives buyers a greater sense of security, which boosts their confidence and lowers product uncertainty [65]. By exhibiting the details of products and their unique selling points, live streaming provides extensive and valuable information for consumers to assess the value of the product and the usefulness of the product recommendation. In other words, sense of telepresence can influence the impact of consumers’ perceived value on perceived credibility. Hence, we propose the following hypothesis:

Hypothesis 3 (H3). The relationship between perceived value and perceived credibility is positively moderated by sense of telepresence.

### Mediating effect of perceived credibility

Perceived credibility refers to the extent to which a message is seen as credible, truthful, or factual [66]. Consumers’ decision-making process is heavily influenced by perceived credibility. An individual who perceives a message to be credible has more confidence in adopting it and using it to make future decisions [67]. Perceived credibility plays an especially prominent role when individuals try to adopt information from online sources since there exists a lot of uncertainty in virtual spaces [68]. Customers generally believe shopping online is riskier than shopping in physical stores [69]. Customers may get information different from what they expect in the online environment, thereby making wrong purchase decisions.

In traditional e-commerce, merchants may manipulate product reviews in order to increase sales. About 15% of online product reviews are generated by direct or indirect manipulation by merchants [7]. As consumers become increasingly aware of the existence of fake product reviews and other kinds of fraudulent product information, they consequently pay more attention to the authenticity and credibility of online product information.

Live streaming has made it possible for consumers to interact in real-time with product information senders and visualize real products, thereby reducing the uncertainty of a virtual transactional environment [70]. One of the advantages of live streaming is that it makes possible close interaction between viewers and live streamers. In traditional e-commerce, customers must leave the product page of online stores to enter the chat interface if they have questions about a product [65]. By contrast, live streaming offers audiences a live chatroom where they may communicate with broadcasters and other viewers in real-time. Broadcasters can respond rapidly and provide audiences with highly individualized advice and services once they ask any questions in the chat room or even through "lianmai" to have a voice chat [71]. Live streaming also enables streamers to display products from various angles and demonstrate how to use these products to assist buyers in obtaining true, dynamic, and extensive product information [46]. The authenticity and visualization of product introductions in live streaming can help customers alleviate worries and anxieties [71].

Perceived credibility is a crucial impetus for overcoming barriers of perceived risk [72] and can lead to positive feelings toward product information [70]. When information is considered credible, it is more likely to be accepted by the recipients and has an impact on their perceptions and behaviors [73]. Consumers are willing to adopt the information to assist decision-making when they believe it to be credible. They also spend less time looking for other sources of information in order to lower their cognitive cost [74]. When consumers obtain more detailed information about products, they can better understand the value of product recommendations. The more selling points the viewers perceive, the more positive their attitudes will be [59]. Consumers will not use the information to help them make decisions unless they are convinced that the information they have received is credible [75, 76]. Based on the above, we propose:

Hypothesis 4 (H4). The relationship between perceived value and intention to adopt product recommendations from live streamers is mediated by perceived credibility.

### Moderating role of self-identification with streamers

Self-identification is grounded in self-congruity theory, which explains the match between the image of an object and the self-concept of consumers [77]. Self-identification refers to the extent to which an individual perceives that an object is reflective of his or her personality [78]. When individuals identify themselves with an object, they develop an emotional bond with it by automatically expressing favoritism for it [79]. A person’s activities are driven by goals, which are organized around self-identifications to which the person is committed [80]. Individuals are motivated to behave in accordance with their identity standards, and thus aspire to a congruence between their self-concept and their enacted behaviors [81]. When particular identities are activated, individuals engage in self-verification processes to evaluate this congruence, which, in turn, increases their sense of control [82].

One may identify with others because of their views, attitudes, erudition, special talents, or even personal charisma [83]. When other individuals exhibit qualities that one could identify with, he or she may develop a sense of intimacy and incorporate others’ characteristics into his or her sense of self [84]. Identification with others sometimes refers to the process by which one puts himself or herself in the place of others and participates in others’ experiences during a program [84]. When watching live streaming, viewers would find product recommendations adoptable when they form a strong self-streamer connection. The self-verification process leads viewers to become emotionally attached to the verifying actor, deepening the viewers’ commitment to the relationship [81]. Following the streamer’s advice is a way to confirm the conceptions of their selves; that is to say, to maintain or enhance a complete and true sense of self [85].

Lam indicated that consumers meet their self-continuity needs via their perceptions of congruence or harmony during their shopping process [86]. Consumers naturally prefer to internalize their product’s purchasing process by meeting their core psychological needs and developing their own self-identity [87]. The desire to express their own self and verify their self-identity frequently serves as the primary motivator that instigates consumers’ behavioral intentions [77]. When individuals’ actions fail to validate or reinforce their self-concept, they would be in a state of discomfort, disorientation, or psychological disorder [88]. Therefore, people are more likely to engage in role-congruent behaviors in order to reinforce their self-identities [89]. To avoid psychological dissonance, individuals search for situations that are consistent with their sense of self and avoid situations that threaten their existing self-concept [90]. Accordingly, we believe that self-identification has a positive impact on consumers’ behavioral intentions. Also, self-identification would influence the effects of other variables on individuals’ behavioral intentions [91], and the level of self-identification would affect individuals’ level of trust and the mechanisms by which trust acts on other variables [92]. Hence, we anticipate that self-identification would play positive moderating roles in the relationships between individuals’ product information adoption intention and its antecedents. When individuals have a high level of self-identification with streamers, they are more likely to develop emotional connections with the streamers [79]. We believe that the emotional connections will influence viewers’ perception of the risks in adopting information from streamers, as well as the mechanism through which other variables affect viewers’ information adoption intention. That is, when individuals have a high level of self-identification with streamers, the positive effect of perceived credibility on their willingness to adopt product information from the streamers would be strengthened. Similarly, we also anticipate that these emotional connections will positively influence the impact of viewers’ perceived value on their intention to adopt product recommendations. Specifically, high levels of identification will amplify the positive effect of perceived value on customers’ adoption intention, and vice versa. Hence, we propose:

Hypothesis 5 (H5). The relationship between perceived credibility and intention to adopt product recommendations is positively moderated by self-identification with streamers.
Hypothesis 6 (H6). The relationship between perceived value and intention to adopt product recommendations is positively moderated by self-identification with streamers.

Gender could play a role in affecting consumers’ evaluative judgments [93]. Men and women seem to weigh variables of product information differently when evaluating the information. Therefore, this study controls for gender in the analyses. Considering other control variables (i.e., age, frequency of online shopping, and frequency of watching live streaming) that may influence consumer intention to adopt product recommendations, we also use them as control variables in this study.

**Fig 1:**
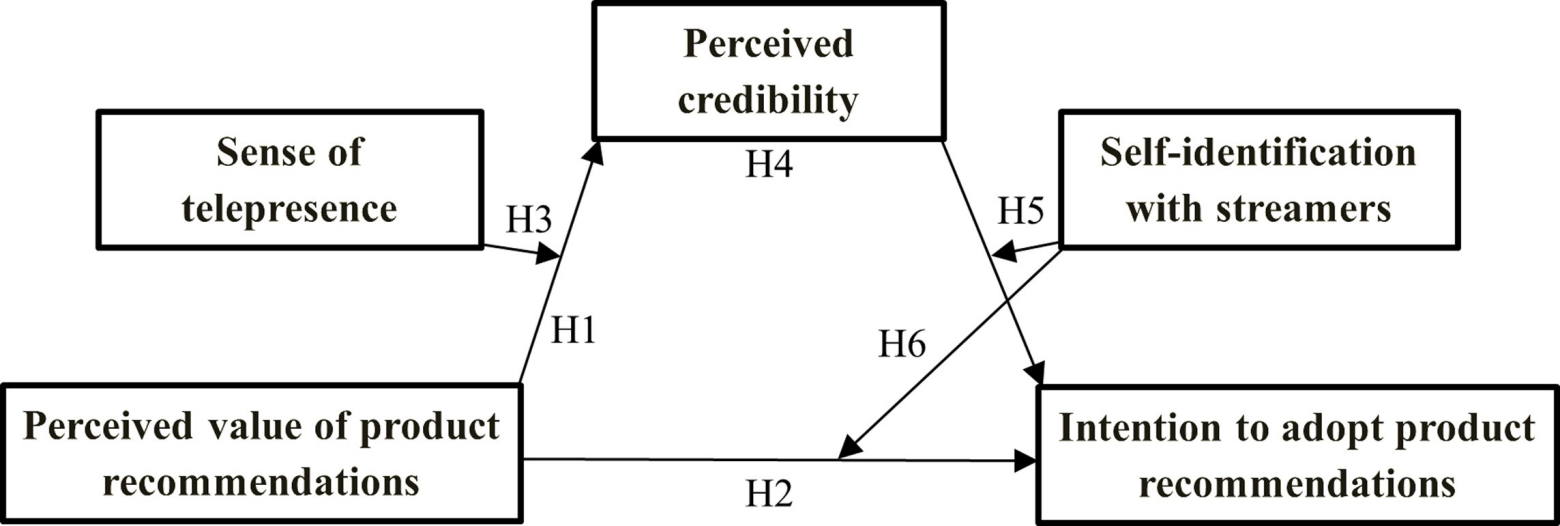
Research model.

Based on the discussion above, we constructed our research model (see Fig 1).

## Materials and methods

### Sample and procedure

Before being launched through wjx.com, a well-known online survey platform in China, the questionnaire was verified by methods of observation and in-depth interviews. First, we watched the live streaming of some well-known live streamers on Douyin and other platforms and even participated in the interaction with streamers in order to check how effectively the variables can reflect real situations. Then several viewers who watched live streaming frequently were invited to check how effectively the questions can reflect the variables. Some improper expressions were modified accordingly.

The formal online survey was conducted in January 2023. All members were recruited from members of wjx.com. Participation was voluntary and user consent was obtained before the start. According to the rules of ethics committees of Fujian Jiangxia University, no formal ethics approval was required in this particular case based on the following reasons: (1) the research does not involve medical experiments, animal experiments, or other experiments that raise ethical concerns. Also, no medical information was collected in this study; (2) the research is of minimal risk to participants, and all necessary precautions have been taken to ensure the safety and well-being of individuals involved. (3) no private information will be published. The primary data used for the current study were collected by using the structured questionnaire, which consists of three sections. The first section includes the basic information of the questionnaire, such as the purpose of the survey, and one screening item, "have you ever watched live streaming? " Samples with an answer of "no" to this question will be excluded as invalid questionnaires. Respondents were asked to answer questions according to their most recent experience of watching live streaming. Confidentiality and anonymity were assured to all participants. The second section encompasses the demographic characteristics of the respondents, including their gender, age, frequency of online shopping, and frequency of watching live streaming. The third section contains the five variable scales of the research model, which have 20 question items.

After excluding the unqualified questionnaires, we have 382 valid questionnaires. The sample consisted of 175 males (45.8%) and 207 females (54.2%). Most were aged between 19 and 40 years, accounting for 69.1%. While 49.7% shop online less than 4 times a week, 50.3% shop online more than 3 times a week. Most respondents watch live streaming for more than 30 minutes per week, accounting for 65.2%, while 34.8% of respondents watch live streaming for less than 30 minutes per week.

### Measures and regression methods

The constructs used in this study were measured using multiple-item scales adopted from previous studies (see S1 Table). Relevant items were revised slightly to align with the context of this study. We have five variables in the main part of the questionnaire, namely, perceived value of product recommendations, perceived credibility, intention to adopt product recommendations, sense of telepresence, and self-identification. All these items were asked on a 5-point Likert scale from 1 (strongly disagree) to 5 (strongly agree).

To improve the validity of the survey results, we deleted questionnaires with the same answer and those with less than 1 minute of answer time. Finally, 382 valid questionnaires were obtained.

In this study, we employed PROCESS macro designed by Hayes [94] to test the hypotheses. Specifically, Model 4 of the PROCESS macro was utilized to examine both direct and indirect effects, while Model 28 was applied to assess the moderated mediation effects. Compared with other regression approaches such as structural equation modeling (SEM), PROCESS macro can better handle complex models with moderator and mediator variables [95]. PROCESS macro offers a more robust approach to testing the size and significance of an effect by using a nonparametric bootstrapping procedure [96]. By PROCESS macro, no assumptions regarding the sampling distribution of the variables are needed and it can be applied to regression analysis with small samples [97]. When using the analysis method of PROCESS macro, the sample size of 295 could produce reliable estimates [98]. The sample size of this study is 382, indicating that it is adequate to fulfill the minimum requirements of sample size for the PROCESS macro model.

## Results

### Non-response bias and common-method bias (CMB)

In order to inspect potential biases in the self-reported survey data, tests for non-response bias and CMB were conducted. Non-response bias was checked by t-test analysis of the data sets of the early and late respondents. Following the suggestion provided by Armstrong and Overton [99], we partitioned the data into early and late periods in a 70:30 ratio. The results of the analysis revealed all p-values of the variables are above 0.2, which exceeds the standard that the p-value should be less than 0.05. That is to say, no substantial differences existed in the means for the two groups of the responses and therefore we can conclude that the data were free of non-response bias.

Although mature scales were utilized in this study, it was not possible to entirely avoid the issue of data source identity, which could potentially result in the presence of CMB. To assess the possible impact of CMB, we conducted three statistical analyses. First, Harmon one-factor test was conducted. Should a single item exhibit a total variance exceeding 50%, it has the potential to introduce common method bias (CMB) into the data, thereby impacting the empirical findings [100]. Results showed that the unrotated first factor accounted for 31.8% of the variance which is within the recommended threshold value of 50%. Second, we conducted an analysis of inner VIF (Variance Inflation Factor) values of the variables in the model. A VIF value exceeding 3.3 is recognized as an indicator of collinearity and potential contamination by CMB [101]. The results showed that all values of VIF in our study were below 2.3, indicating that CMB-related problems were not present in our data. Third, we utilized the common latent factor method by introducing a latent variable that was loaded by all survey items [102]. The standardized regression weights were then compared between models with and without this common latent variable. The findings revealed that the differences in weights did not surpass 0.1. The result also suggests that CMB should not be an issue for this data set [102].

### Reliability and validity

We examined the model’s reliability and validity by using SPSS 24 and Amos 21. As Table 2 shows, Cronbach’s alpha for all constructs varied from 0.755 to 0.899, which obviously meets the recommended threshold value of 0.70 [103]. The composite reliability values were all greater than the suggested threshold value of 0.7 [103], ranging from 0.759 to 0.899. Consequently, all constructs in the model exhibit acceptable reliability.

To assess convergent validity, we followed the method recommended by Fornell and Larcker [104]. As we can see in Table 1, all AVEs of the constructs were significantly above the threshold value of 0.50 suggested by Fornell and Larcker. In addition, factor loadings of all items were larger than 0.705, which exceeded the acceptable level of 0.70. It was indicated that our instruments exhibited acceptable convergent validity.

**Table 1:** Reliability and validity of the constructs.

Construct	Items	Factor loadings	Cronbach’s α	Composite reliability	Average variance extracted
Perceived Value	PV1	0.763	0.859	0.861	0.609
	PV2	0.796			
	PV3	0.848			
	PV4	0.708			
Telepresence	TP1	0.734	0.899	0.899	0.641
	TP2	0.810			
	TP3	0.828			
	TP4	0.831			
	TP5	0.797			
Perceived Credibility	PC1	0.734	0.825	0.826	0.542
	PC2	0.756			
	PC3	0.720			
	PC4	0.735			
Self-identification	SI1	0.764	0.882	0.887	0.665
	SI2	0.863			
	SI3	0.884			
	SI4	0.740			
Adoption Intention	AI1	0.705	0.755	0.759	0.512
	AI2	0.736			
	AI3	0.705			

Furthermore, we assessed discriminant validity by the criterion proposed by Fornell and Larcker [104] and by the heterotrait-monotrait ratio (HTMT). Fornell and Larcker believed that the potential variable correlation coefficient should be less than the square root of its AVE value [104]. As is shown in Table 2, none of the potential variable correlation coefficients is greater than the square root of its AVE value. Thus, we can conclude discriminant validity.

**Table 2:** Descriptive statistics, correlation matrix, reliability, and square root of AVE.

Construct	M	SD	1	2	3	4	5
1.Perceived Value	3.310	0.687	**0.780**				
2.Telepresence	3.819	0.739	0.008	**0.801**			
3.Perceived Credibility	3.346	0.577	0.614**	0.432**	**0.736**		
4.Self-identification	2.977	0.803	0.100	0.111*	0.144**	**0.815**	
5.Adoption Intention	3.374	0.522	0.546**	0.257**	0.543**	0.532**	**0.716**

Note:

*p < 0.05

**p < 0.01.

The diagonal data is the square root of the latent variable AVE value, and the font is bold.

Under the HTMT approach, if the values of HTMT are less than 0.85, the validity of the constructs with respect to discrimination is acceptable [105]. Table 3 displays the results of the HTMT test, all of which fall below the recommended Kline threshold of 0.85. Besides, both the lower and upper confidence intervals (CIs) do not include the value of 1, confirming the discrimination of the constructs.

**Table 3:** Heterotrait-monotrait ratio.

Construct	1	2	3	4	5
1.Perceived Value					
2.Telepresence	0.07 CI_0.95_ [0.06, 0.154]				
3.Perceived Credibility	0.728 CI_0.95_ [0.655, 0.796]	0.501 CI_0.95_ [0.415, 0.583]			
4.Self-identification	0.112 CI_0.95_ [0.059, 0.23]	0.131 CI_0.95_ [0.068, 0.234]	0.168 CI_0.95_ [0.069, 0.276]		
5.Adoption Intention	0.677 CI_0.95_ [0.596, 0.754]	0.314 CI_0.95_ [0.21, 0.425]	0.688 CI_0.95_ [0.6, 0.771]	0.654 CI_0.95_ [0.563, 0.747]	

### Mediation analyses

In H1 and H2, we assume that perceived value would positively affect perceived credibility and will positively influence individuals’ product recommendation adoption intention. In H4, we propose that perceived credibility would mediate the relationship between perceived value and adoption intention. We employed PROCESS macro (Model 4) to test the hypotheses above. First, in model 1, we found that perceived value was positively associated with perceived credibility (β = 0.498, t = 13.696, p < 0.001) as shown in Table 4. Then we found that perceived value could significantly predict adoption intention (β = 0.405, t = 11.694, p < 0.001) by testing model 2. When perceived value and perceived credibility were taken together as predictors, both perceived value (β = 0.257, t = 6.368, p < 0.001) and perceived credibility (β = 0.298, t = 6.367, p < 0.001) can significantly affect adoption intention. In order to examine the indirect effects of perceived value further, we employed bootstrapping based on the 95% bias-corrected confidence interval (CI). The results showed that the indirect effect was also positively significant (indirect effect = 0.148, 95% CI = [0.099, 0.204]), and the ratio of indirect to the total effect of perceived value on adoption intention was 36.5%. Therefore, perceived value plays a partial mediating role in the association between perceived value and adoption intention. To sum up, H1 and H2 are both supported by the data while H4 is partially supported.

**Table 4:** Testing perceived credibility as a mediator in the relationship between perceived value and adoption intention.

Construct	Model 1(PC)		Model 2(INT)		Model 3(INT)	
	*β*	*t*	*β*	*t*	*β*	*t*
Gender	0.149	3.062**	0.014	0.307	-0.030	-0.675
Age	0.275	1.105	0.101	4.238***	0.092	4.086***
FS	-0.015	-0.778	0.006	0.345	0.011	0.618
FW	-0.021	-1.131	-0.003	-0.199	0.003	0.162
PV	0.498	13.696***	0.405	11.694***	0.257	6.368***
PC					0.298	6.367***
*R* ^*2*^	0.396		0.331		0.396	
*F*	49.235***		37.124***		40.948***	

Note:

*p < 0.05

**p < 0.01

***p < 0.001.

FS, frequency of online shopping; FW, frequency of watching live streaming; PV, perceived value; PC, perceived credibility; INT, intention to adopt product recommendations.

### Moderation analysis

In H3, we propose that sense of telepresence could moderate the link between perceived value and perceived credibility. In H5 and H6, we assume that self-identification could moderate the link between perceived credibility and adoption intention, and the association between perceived value and adoption intention. After adjusting for factors including gender, age, frequency of online shopping, and frequency of watching live streaming, we performed a moderated mediation analysis by using Hayes’ PROCESS macro (Model 28). In each model, we controlled for viewers’ gender, age, frequency of online shopping, and frequency of watching live streaming. The results are shown in Table 5. In model 4, both perceived value (β = 0.485, t = 13.888, p < 0.001, 95% CI = [0.416, 0.554]) and sense of telepresence (β = 0.334, t = 11.789, p < 0.001, 95%CI = [0.278, 0.389]) were positively associated with perceived credibility, but the interaction effect of perceived value and sense of telepresence on perceived credibility was not significant (β = -0.031, t = -0.704, p > 0.05, 95% CI = [-0.118, 0.056]). In model 5, perceived credibility (β = 0.409, t = 12.046, p < 0.001, 95% CI = [0.343, 0.476]) and self-identification (β = 0.298, t = 12.586, p < 0.001, 95% CI = [0.252, 0.345]) were both positively related to adoption intention. Moreover, the interaction effect of perceived credibility and self-identification (β = 0.111, t = 2.777, p < 0.01, 95% CI = [0.032, 0.189]) on adoption intention was also significant. In model 6, it was found that perceived value (β = 0.242, t = 7.335, p < 0.001, 95% CI = [0.177, 0.307), perceived credibility (β = 0.250, t = 6.487, p < 0.001, 95% CI = [0.174, 0.325]) and self-identification (β = 0.287, t = 12.879, p < 0.001, 95% CI = [0.243, 0.330]) were all positively associated with adoption intention; the interaction effect of perceived credibility and self-identification (β = 0.190, t = 3.895, p < 0.001, 95% CI = [0.094, 0.286]) on adoption intention was significant; the interaction effect of perceived value and self-identification on adoption intention was also significant, but the coefficient was negative (β = -0.106, t = -2.520, p < 0.05, 95% CI = [-0.189, -0.023]). Since H6 states that "the relationship between perceived value and intention to adopt product recommendations is positively moderated by self-identification", H6 was also not supported. In summary, both H3 and H6 are not supported by the data while H5 is supported.

**Table 5:** Testing perceived credibility as a mediator and sense of telepresence and self-identification as two moderators in the relationship between perceived value and adoption intention.

Construct	Model 4(PC)		Model 5(INT)		Model 6(INT)	
	β	t	β	t	β	t
Gender	0.110	2.660**	0.011	0.288	-0.014	-0.377
Age	0.024	1.149	0.053	2.590*	0.048	2.251*
FS	-0.012	-0.742	-0.004	-0.227	0.005	0.342
FW	-0.011	-0.717	0.029	2.082*	0.005	0.398
PV	0.485	13.888***			0.242	7.335***
TP	0.334	11.789***				
PV×TP	-0.031	-0.704				
SI			0.298	12.586***	0.287	12.879***
PC			0.409	12.046***	0.250	6.487***
PV×SI					-0.106	-2.520*
PC×SI			0.111	2.777**	0.190	3.895***
R^2^	0.570		0.531		0.600	
F	70.769***		60.590***		61.988***	

Note:

*p < 0.05

**p < 0.01

***p < 0.001.

FS, frequency of online shopping; FW, frequency of watching live streaming; PV, perceived value; TP, sense of telepresence; PC, perceived credibility; SI, self-identification; INT, intention to adopt product recommendations.

**Fig 2:**
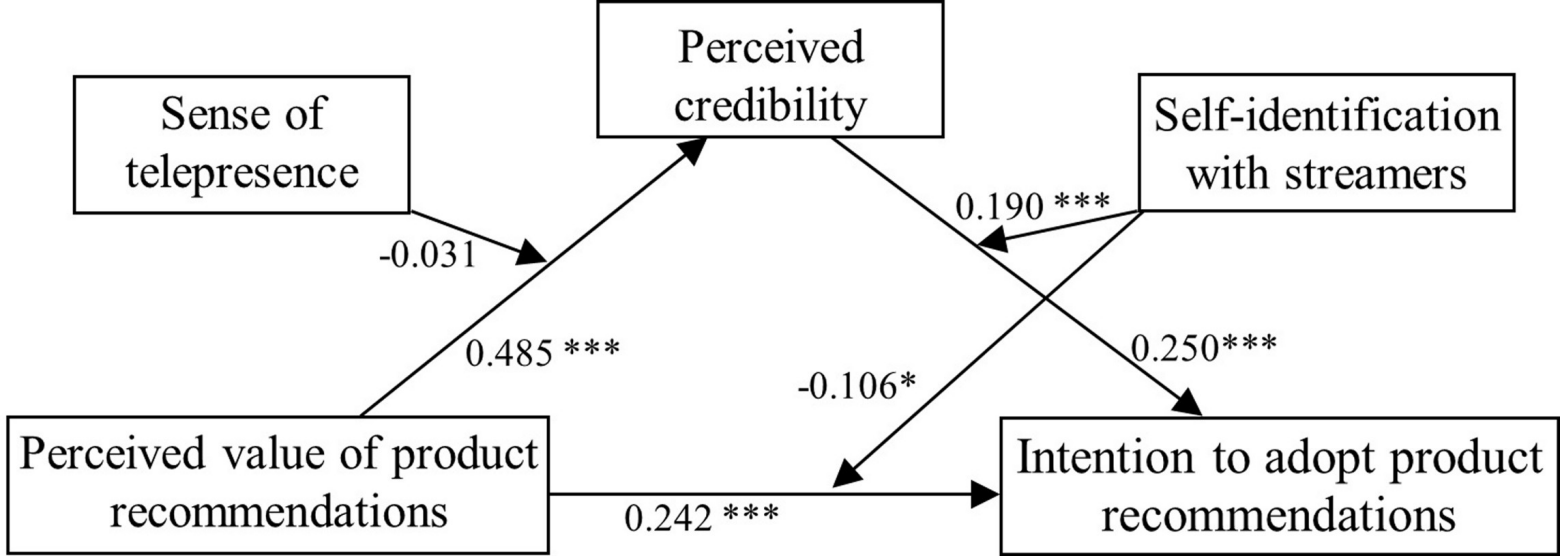
Model of the moderating roles of sense of telepresence and self-identification on the direct and indirect relationship between perceived value of product recommendations and intention to adopt product recommendations.

Fig 2 shows the whole picture of the effects of the moderating roles of sense of telepresence and self-identification, as well as the direct and indirect effects of perceived value on customers’ intention to adopt product recommendations from streamers.

**Fig 3:**
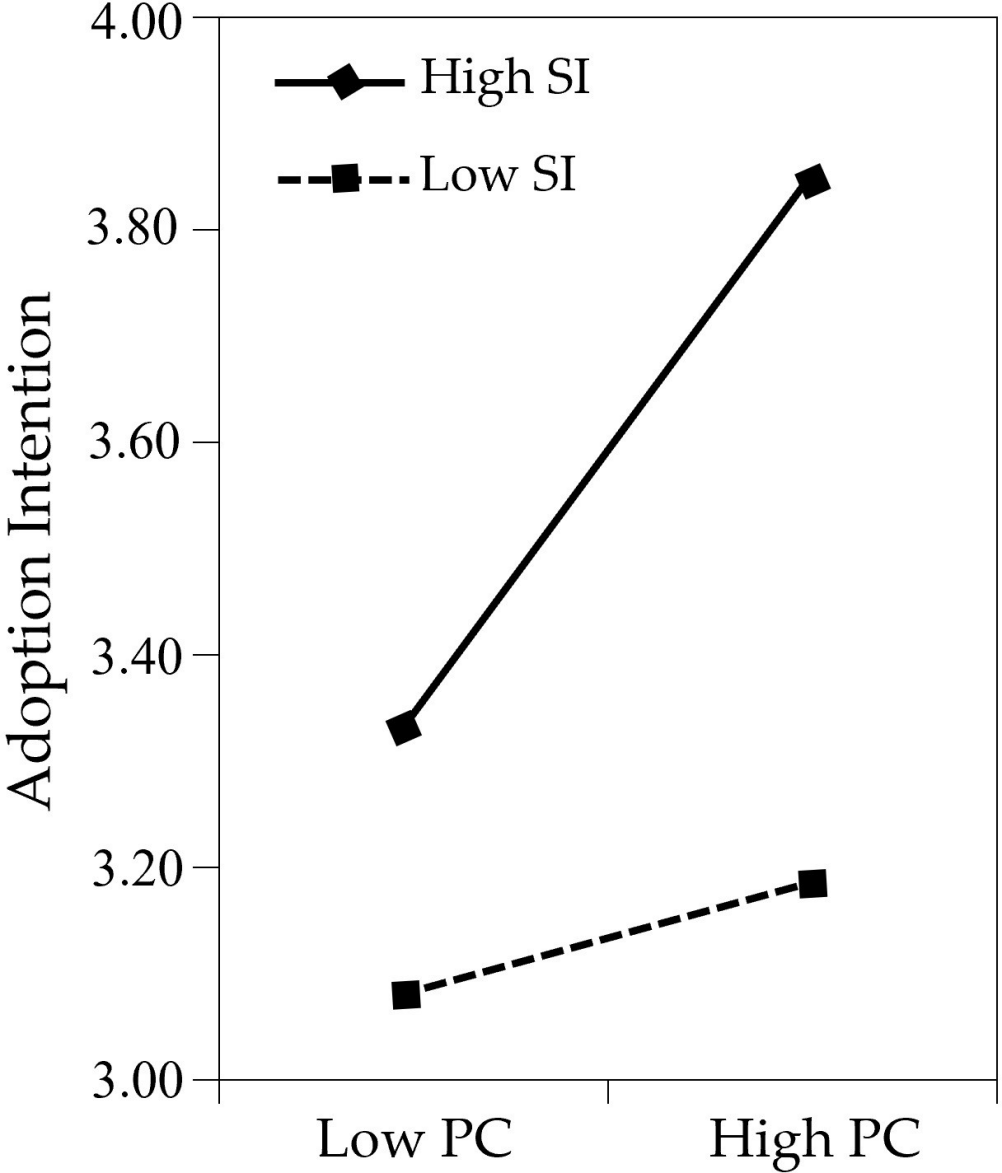
Moderating effect of SI on PC-INT.

**Fig 4:**
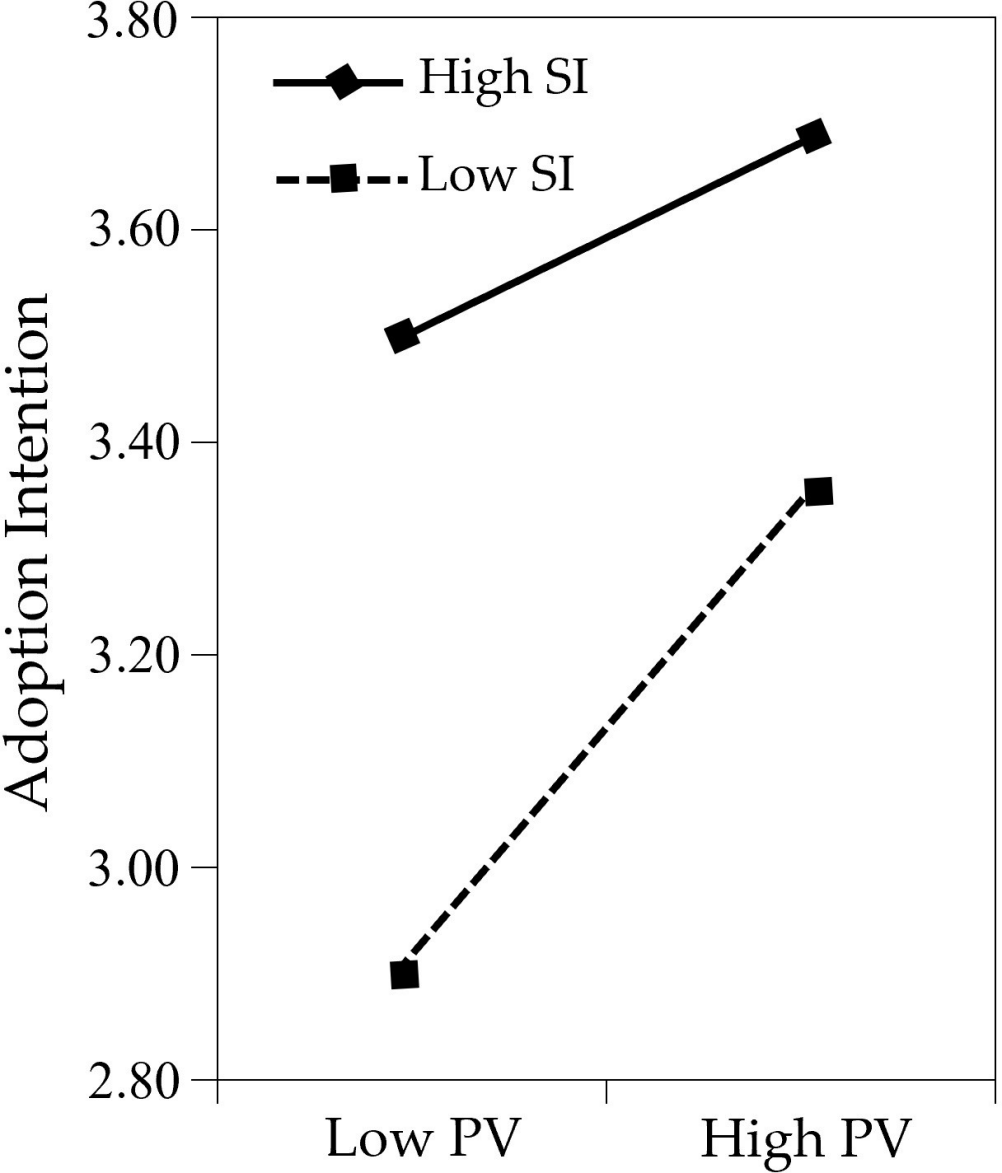
Moderating effect of SI on PV-INT.

For descriptive purposes, the nature of the moderation was further explored by using a simple slope analysis. First, we predicted adoption intention against perceived credibility, separately for low, medium, and high levels of self-identification (see Fig 3 and Table 6). Simple slope tests showed that perceived credibility could significantly predict adoption intention when viewers had high levels of self-identification (β_simple_ = 0.402; t = 7.260; p < 0.001) and medium levels of self-identification (β_simple_ = 0.250; t = 6.487; p < 0.001). In contrast, this relation became statistically nonsignificant for viewers with low levels of self-identification (β_simple_ = 0.097; t = 1.779; p = 0.076). These results indicated that the relationship between perceived credibility and adoption intention was strengthened when self-identification was high. Then we predicted adoption intention against perceived credibility, separately for low, medium, and high levels of self-identification. The results are shown in Fig 4 and Table 7. Simple slope tests showed that perceived value could significantly predict adoption intention when viewers had high levels of self-identification (β_simple_ = 0.157; t = 3.187; p < 0.01), medium levels of self-identification (β_simple_ = 0.242; t = 7.335; p < 0.001), and low levels of self-identification (β_simple_ = 0.328; t = 7.240; p < 0.001). As shown in Fig 3(B), in situations of high self-identification (illustrated by the solid line), the level of adoption intention tends to be higher. As shown in Fig 3(B), the slope is steeper when self-identification is low (dotted line), whereas the slope is flat when self-identification is high (solid line). These results indicate that the positive relationship between perceived value and adoption intention is weakened when self-identification is at a high level.

**Table 6:** The impact of perceived credibility on adoption intention at different levels of self-identification.

Self-identification	B	SE	t	p	Boot LLCI	Boot ULCI
M-1SD	0.097	0.054	1.779	0.076	-0.010	0.204
M	0.250	0.039	6.487	0.000	0.174	0.325
M+1SD	0.402	0.055	7.260	0.000	0.293	0.511

Note: LL, low limit; CI, confidence interval; UL, upper limit; M, mean; SD, standard deviation

Note: *p < 0.05, **p < 0.01, ***p < 0.001.

**Table 7:** The impact of perceived value on adoption intention at different levels of self-identification.

Self-identification	B	SE	t	p	Boot LLCI	Boot ULCI
M-1SD	0.328	0.045	7.240	0.000	0.239	0.417
M	0.242	0.033	7.335	0.000	0.177	0.307
M+1SD	0.157	0.049	3.187	0.002	0.060	0.254

Note: LL, low limit; CI, confidence interval; UL, upper limit; M, mean; SD, standard deviation

## Discussion and conclusion

### Discussion

This study aims to understand the mechanism behind individuals’ intention to adopt product recommendations in the live-streaming environment. To that end, we implemented a moderated mediation model to test the direct and indirect effects of perceived value on adoption intention, the moderating role of sense of telepresence in the link between perceived value and perceived credibility, and the moderating roles of self-identification in the associations between perceived credibility and adoption intention, and between perceived value and adoption intention.

First of all, we examined the relationship between perceived value and users’ intention to adopt product recommendations. Previous studies have shown that perceived value could positively influence customers’ behavioral intentions in various contexts, such as tourism management [106] and environmental protection [107]. Consistent with previous studies [48, 106, 107], we discovered a positive relationship between perceived value and users’ intention to adopt product recommendations (β = 0.405, p < 0.001). This finding extends the research on the impact of perceived value on customers’ behavioral intention to the context of live streaming, demonstrating the crucial role of perceived value in consumer behavior decision-making in the online environment, and illustrating the validity of the principle of rationality in the customers’ adoption of online information.

We then explored the association between perceived value and perceived credibility. We found that perceived value could significantly influence perceived credibility (β = 0.498, p < 0.001). When individuals can perceive the economic value and other kinds of values of the product recommendation, they will be more likely to develop a positive attitude towards the product recommendation; that is, they will assess the product recommendation more favorably and tend to believe it. Previous studies have confirmed the significant relationship between perceived value and perceived credibility [108]. This study advances previous research by offering empirical data on live-streaming themes, highlighting the crucial role of perceived value in shaping individuals’ online attitudes, and clarifying the mechanisms of perceived credibility formation online.

By examining the moderating role of sense of telepresence on the association between perceived value and perceived credibility, we found that perceived value (β = 0.485, p < 0.001) and sense of telepresence (β = 0.334, p < 0.001) could positively affect perceived credibility independently. However, when the sense of telepresence interacts with perceived value, their interaction does not significantly affect perceived credibility (β = -0.031, p > 0.05). This differs from earlier findings, which propose that a sense of telepresence, when interacting with perceived value, can affect viewers’ perceived credibility [64, 65]. These studies suggest that individuals who experience a stronger sense of telepresence may have a clearer understanding of the product information’s value. As a result, their uncertainty will decrease, leading to greater confidence in trusting the product information. Unexpectedly, our study found that the moderating effect of the sense of telepresence is not significant. One possible explanation is that although sense of telepresence may help viewers better understand the value of product recommendations, perceived credibility for consumers is primarily influenced by the perceived value of the recommendations themselves. In other words, sense of telepresence may not effectively strengthen or weaken the effects of perceived value on perceived credibility.

The analysis results also show that the relationship between perceived credibility and individuals’ intention to adopt product recommendations is positive (β = 0.298, p < 0.001). This aligns with previous studies which suggest the predicting role of perceived credibility on individuals’ behavioral intention [73, 75, 109]. Also, we examined the mediating role of perceived credibility and found that the total effect, the indirect effect, and the direct effect of perceived value on adoption intention are all supported by the results. The ratio of indirect to the total effect of perceived value on adoption intention was 36.5%. Consequently, the partial mediating role of perceived credibility was supported. The result indicates the vital role of perceived credibility in viewers’ intention to adopt product recommendations, lending strength to the credibility theory, which highlights its positive impact on information adoption [110].

The effects of self-identification on users’ intention to adopt product recommendations were also explored in this study. The results show that self-identification could positively moderate the relationship between perceived credibility and viewers’ intention to adopt product recommendations (β = 0.190, p < 0.001). That is to say, when the level of viewers’ self-identification is high, the effect of perceived credibility on individuals’ adoption intention will be stronger. This result further validates the crucial role of perceived credibility in users’ behavioral intention in the live-streaming context. We also found that self-identification could significantly moderate the link between perceived value and users’ intention to adopt product recommendations. Unexpectedly, the coefficient was negative (β = -0.106, p < 0.05). A possible explanation is that there exists a substitution effect between perceived value and self-identification. When self-identification is at a low level, consumers’ intention to adopt product recommendations primarily depends on the value they perceive. However, when the level of self-identification is relatively high, the perceived value of product recommendations becomes less important to viewers when deciding whether to adopt the recommendations, compared to when self-identification is low. In other words, the influence of self-identification can attenuate the effect of perceived value on adoption intention. Previous research has identified the positive moderating effect of self-identification [91]. The results of this study extend these findings and further reveal the complex moderating role of self-identification in live-streaming commerce context.

### Theoretical contribution

First, this study advances the current literature on customers’ information adoption behaviors in the live-streaming environment. Existing research on consumer behaviors in the live-streaming context primarily focuses on customer behaviors such as impulsive buying [1, 11], gift-giving [18], environmental management [107], etc. Research on the dissemination mechanisms of product information in live streaming is relatively sparse. Considering the distinctive features of product information dissemination patterns in the live-streaming environment, and the significant roles that live streamers play in influencing consumer product choices, it is imperative to delve into the unique mechanisms of consumer information adoption in the live-streaming context.

Second, the findings of this study provide empirical evidence that perceived value and perceived credibility are crucial determinants of users’ intention to adopt information in the context of live streaming. Although previous studies have shown the roles of perceived value and perceived credibility in customers’ purchase intention [38, 49], brand loyalty [44], etc., empirical research on perceived value and perceived credibility has been scarce in the context of information adoption in live streaming. By analyzing data collected from Chinese live-streaming viewers, this study uncovers how perceived value directly and indirectly influences consumers’ willingness to adopt information, as well as how perceived credibility serves as a mediating factor in the relationship between perceived value and product information adoption in live streaming. This illustrates the applicability of the value-intention model in research on consumer online information adoption and contributes to a deeper understanding of the value-intention framework.

Finally, given the actual circumstances of consumer information adoption in the live-streaming environment, we introduced two new variables into the value-intention framework: sense of telepresence and self-identification with streamers. Unlike previous literature, the results indicate that the sense of telepresence did not have a significantly moderating influence on the relationship between perceived value and perceived credibility, which provides a new perspective on our understanding of the role of sense of telepresence in consumer behavior. At the same time, the analysis results show that the moderating effects of self-identification on the relationships between perceived value and adoption intention, as well as between perceived credibility and adoption intention, are significant. These results could lend strength to findings of previous studies that individuals’ self-identification needs could affect their decision-making process [89], and consumers’ drive to express their selves and verify their self-identity was a crucial motivator propelling their behavioral intentions [90]. Also, this study extends the self-identity theory [111] to social commerce context and provides empirical evidence for the crucial moderating role of self-identification in consumers’ online decision-making process.

### Practical implications

First, the results of this study show that perceived value is a key antecedent variable that encourages livestream viewers to adopt product recommendations. As the value-intention framework suggests, individuals evaluate the perceived value of an object by comparing the gains and sacrifices associated with it. When watching a live stream, audiences invest time, energy, and other resources. Consequently, to enhance viewers’ perceived value when watching live streamers’ product recommendations, live streamers should offer their audiences some benefits. For instance, they could recommend products with high cost-effectiveness, share insights on product performance, discuss their personal experiences with the recommended products, and suggest reliable purchasing channels, among other things. Also, since many viewers watch live streams to relax, it is advisable to incorporate entertainment elements into their live-streaming programs. In this way, live streamers’ product recommendations will be better adopted.

Second, we found that sense of telepresence, together with perceived value, could positively predict perceived credibility. Traditionally, it is almost impossible for customers to experience all the details of the products they want to purchase online, but live streaming enables customers to experience product features in an interactive way. As a result, live streamers should make the most of live streaming to present product details from various angles and engage in real-time interaction with their audience during their live broadcasts. Also, the study showed the importance of perceived credibility which plays a partial intermediary role in the association between perceived value and adoption intention. Consequently, live streamers should prioritize the credibility of the product information in order to enhance the persuasiveness of their product recommendations.

Lastly, our study showed the vital role of self-identification in customers’ adoption intention of product recommendations. As a result, live streamers should find ways to cultivate emotional connections with audiences and work on creating memorable experiences during the process of live streaming, so as to create emotional resonance in viewers and enhance viewers’ sense of self-identification. Moreover, when selecting live streamers to promote their products, social media managers should not make their choices solely based on metrics like the number of followers of streamers and the streamers’ frequency of interactions with followers. More attention should be paid to the emotional connection between streamers and followers, as well as the congruence between live streamers and potential customers.

### Limitations and future research

There exist several limitations to this study that could be addressed in future studies. First, we did not limit the product category of product recommendations in live streaming when collecting data. For instance, the trust mechanisms that influence how viewers accept product recommendations for agricultural products may differ from those that affect their trust in recommendations for cosmetics. Second, although we included the gender of viewers as a control variable, the gender of streamers was not categorized in the survey. Streamers of different genders may show different character traits. The mechanism by which self-identification works may also be differential. Future researchers could undertake relevant studies on the gender of streamers to help better understand the impact of the streamers’ gender on the adoption intention of product recommendation. Third, the data for this study was collected from live-stream viewers in China. Given that people from different regions and countries have diverse cultures and preferences, the mechanisms driving customers’ intention to adopt product recommendations may vary as well. Future researchers could gather data from live-stream viewers in various regions and countries to conduct comparative studies on the psychological mechanisms underlying product recommendation adoption among customers with different cultural backgrounds and preferences.

## Supporting information

S1 Data (RAR)

S1 Table Measurement items. (DOCX)
