# Supplementary material for: Exploring the underlying mechanisms of customers’ intention to adopt product recommendations from live streamers: A moderated mediation approach
Source: PLoS One. 2025 Feb 13;20(2):e0314682. doi: 10.1371/journal.pone.0314682 (PMC11825015; doi:10.1371/journal.pone.0314682)
Supplement: S1 Table — (DOCX) [file pone.0314682.s002.docx]

**S1 Table.** Measurement items.

| **Measurement Dimension** | **No.** | **Items** | **Reference Source** |
| --- | --- | --- | --- |
| Perceived value | PV1 | I purchase satisfactory products by following product recommendations of streamers at a reasonable price | Chiang et al. and Kuo et al. [1,2] |
|  | PV2 | Purchasing products by following product recommendations of streamers is worth the sacrifice of time and effort |  |
|  | PV3 | Compared with other ways, it is wise to choose the product recommended by streamers for purchases |  |
|  | PV4 | Overall, purchasing products by following product recommendations of streamers makes me feel good |  |
| Telepresence | TP1 | While watching live streaming, I felt like I was in the new world that live streaming was creating | Pelet et al. [57] |
|  | TP2 | I forget my immediate environment when I watch live streaming |  |
|  | TP3 | While I watch live streaming, I feel like my body is in the room, but my mind is inside the world created by the website |  |
|  | TP4 | The products shown in live streaming were so real and three-dimensional that they seemed to be right in front of me |  |
|  | TP5 | The live streaming-generated world seems to me "somewhere I visit" rather than "something I see" |  |
| Perceived credibility | PC1 | I do believe that the product recommendation of the streamer is convincing | Chetioui et al. [3] |
|  | PC2 | I do believe that the product recommendation of the streamer is credible |  |
|  | PC3 | I do believe that the product recommendation is a good reference for purchasing products |  |
|  | PC4 | I find purchasing product by following the product recommendation of the streamer to be worthwhile |  |
| Self-identification | SI1 | I identify with the live streamer | Helm et al. [4] |
|  | SI2 | When I talk about this streamer, I usually say "we" rather than "they" |  |
|  | SI3 | I feel that my personality and the personality of this streamer are very similar |  |
|  | SI4 | I have a lot in common with other followers of the streamer |  |
| Adoption intention | INT1 | I always agree with the content of product recommendation of the streamer | Sussman and Siegal [67] |
|  | INT2 | I will follow the suggestion of the product recommendation again |  |
|  | INT3 | The product recommendation from the streamer always motivates me to make purchase decisions |  |

# References

Chiang CH, Tseng KC. The influence of fan pages on consumer purchase intention: Liking behavior as a moderator. *Journal of Marketing Management.* 2017; 5(2): 44-59. https://doi.org/10.15640/jmm.v5n2a5

Kuo YF, Wu CM, Den WJ. The relationships among service quality, perceived value, customer satisfaction, and post-purchase intention in mobile value-added services. *Computers in Human Behavior.* 2009; 25(4): 887-896*.* [https://doi.org/10.1016/j.chb.2009.03.003](https://doi.org/10.1016/j.chb.2009.03.003)

Chetioui Y, Benlafqih H, Lebdaoui H. How fashion influencers contribute to consumers' purchase intention. *Journal of Fashion Marketing and Management: An International Journal*, 2020; 24(3): 361-380. [https://doi.org/10.1108/JFMM-08-2019-0157](https://doi.org/10.1108/JFMM-08-2019-0157)

Helm SV, Renk U, Mishra A. Exploring the impact of employees’ self-concept, brand identification and brand pride on brand citizenship behaviors. *European Journal of Marketing.* 2016; 50(1/2): 58-77*.* <https://doi.org/10.1108/EJM-03-2014-0162>
